# Plasma β-amyloid, tau, neurodegeneration biomarkers and inflammatory factors of probable Alzheimer’s disease dementia in Chinese individuals

**DOI:** 10.3389/fnagi.2022.963845

**Published:** 2022-08-18

**Authors:** Qingling Sun, Jingnian Ni, Mingqing Wei, Siwei Long, Ting Li, Dongsheng Fan, Tao Lu, Jing Shi, Jinzhou Tian

**Affiliations:** ^1^Department of Neurology, Dongzhimen Hospital, Beijing University of Chinese Medicine, Beijing, China; ^2^Department of Neurology, Peking University Third Hospital, Beijing, China; ^3^School of Life Sciences, Beijing University of Chinese Medicine, Beijing, China

**Keywords:** Alzheimer’s disease, plasma biomarkers, inflammatory factors, ATN, Simoa

## Abstract

**Background:**

Plasma-derived β-amyloid, tau, and neurodegeneration (ATN) biomarkers can accurately diagnose Alzheimer’s disease (AD) and predict its progression. Few studies have investigated the relationship between plasma biomarkers and changes in plasma inflammatory markers in clinically diagnosed AD.

**Methods:**

Seventy-four participants were recruited, including 30 mild-to-moderate AD dementia patients and 44 normal controls (NC). All participants underwent neuropsychological testing and blood sampling for biomarker testing. AD was clinically diagnosed according to the National Institute on Aging-Alzheimer’s Association (NIA-AA) core criteria and required age-mismatched hippocampal atrophy. We performed Single Molecule Array (Simoa), an ultra-sensitive enzyme-linked immunosorbent assay (ELISA), to examine plasma ATN markers, including β-amyloid (Aβ) 40, Aβ42, p-tau181, total (t)-tau, neurofilament protein light chain (NfL), and inflammatory factors (TNF-α, IL-1β, IL-6, and IL-8).

**Results:**

The level of the plasma Aβ42/Aβ40 ratio was significantly declined and the levels of the plasma p-tau181, NfL and TNF-α were significantly higher in the AD group than the NC group, but there was no significant difference in the levels of plasma t-tau, IL-1β, IL-6, and IL-8 between the AD and NC groups. The levels of plasma p-tau181, NfL, Aβ42/Aβ40 ratio, and TNF-α were all associated with impairments in multiple cognitive domains. Among them, the plasma Aβ42/Aβ40 ratio, and the p-tau181 and TNF-α levels were associated with impairments in global cognition, memory, and visuospatial abilities, but not with executive function, only plasma NfL level was associated with executive function. Plasma NfL showed higher diagnostic performance in AD than in NC individuals (AUC = 0.833). A combined diagnostic prediction model of plasma Aβ42/Aβ40 ratio, p-tau 181, and NfL had the highest value than each factor alone (AUC = 0.902),with a sensitivity and specificity of 0.867 and 0.886, respectively.

**Conclusion:**

The levels of plasma ATN biomarkers (Aβ42/Aβ40 ratio, p-tua181, and NfL) were significantly changed in clinically diagnosed AD patients and they all associated with different domains of cognitive impairment. Plasma ATN biomarkers better differentiate mild-to-moderate AD dementia from NC when they are incorporated into diagnostic models together rather than individually. Plasma ATN biomarkers have the potential to be a screening tool for AD. However, the expression of inflammatory factors in AD patients requires further research.

## Introduction

Alzheimer’s disease (AD) is the most common cause of dementia. The clinical manifestations are mainly the impairment of different cognitive domains such as memory, language, thinking, and executive function, which leads to declined activities of daily living, and is accompanied by neurological and psychiatric symptoms. With the increase in aging in the global population and increasing AD prevalence, AD has become one of the world’s greatest medical, social, and economic challenges (Wang and Ding, 2008). Currently, the number of dementia patients in the world is 50 million, and it is estimated that this number will reach 152 million by 2050 (Alzheimer’s Disease International, 2018).

The β-amyloid (Aβ) hypothesis and tau hypothesis are still the most important pathological hypotheses of AD. Known sporadic AD risk genes, such as APOE, APOJ, and TOMM40, may be directly or indirectly involved in AD pathology by regulating the Aβ or tau pathways (Guo et al., 2017). Although the treatment aimed at reducing Aβ deposition and tau tangle formation has not been shown to be effective by randomized controlled trials, Aβ and tau, as the most classic pathological markers, are key in the biological definition of AD. With the use of magnetic resonance imaging, positron emission tomography (PET) imaging, and cerebrospinal fluid (CSF) assays, a diagnostic scheme was recommended based on biomarker evidence of β-amyloid, tau, and neurodegeneration (ATN) (Jack et al., 2016). In China, CSF testing and PET examination technology for AD are only used in a few teaching hospitals, and has not become a routine clinical examination. An ultra-sensitive detection method enables plasma biomarkers to be used in the clinical diagnosis of AD. Plasma-derived markers related to ATN show a good correlation with AD pathology and can be used as an alternative examination when CSF or PET cannot be used. Studies have shown that blood Aβ40 or Aβ42 levels can be used to assess the formation of amyloid plaques in the brain (Olsson et al., 2016; Pereira et al., 2017). Furthermore, plasma p-tau181 can identify AD pathophysiology with high accuracy (Lantero Rodriguez et al., 2020), because it distinguishes AD and non-AD neurodegeneration is similar to tau-PET and CSF p-tau181 (Janelidze et al., 2020). Herein, we focused on plasma neurofilament protein light chain (NfL), which is a sensitive biomarker of axonal damage. Although it is not an AD-specific biomarker, it can still be used as a non-invasive biomarker associated with neurodegeneration in AD patients (Mattsson et al., 2019). Whether NfL can be included in the “N” in the plasma ATN framework is worth exploring.

It has been established that there is an inflammatory response in the occurrence and development of AD, and that the relationship between inflammatory factors and AD is complex. Although studies have shown that inflammatory responses can mediate cognitive impairment in the early stages of neurodegeneration (Ott et al., 2018), it is unclear whether the inflammatory response is a mere bystander of senile plaque and neurofibrillary tangles activation or a causative factor in AD. Because of the use of different detection technologies, it is controversial whether the inflammatory response in AD is limited to the brain tissue or whether it is a systemic inflammatory response. A recent meta-analysis and systematic review supported the view that AD was associated with peripheral and CSF inflammatory responses (Shen et al., 2019). Inflammatory markers, such as TNF-α, IL-1β, IL-6, and IL-8, which are pro-inflammatory factors released by reactive microglia, have been strongly implicated in neurodegeneration and AD pathology (Khemka et al., 2014). Therefore, this study investigated the levels of plasma biomarkers and plasma inflammatory factors in AD patients simultaneously. As detection techniques improve, the non-invasive quantification of molecules is becoming more feasible. Whether the combination of ATN and plasma inflammatory factors will increase the accuracy remains to be determined.

As mentioned above, the quantitative detection of plasma biomarkers and inflammatory factors shows great promise in determining the pathophysiology of AD. Blood-based protein detection has been a challenge because the blood protein content is lower than that in CSF. The newly developed Single Molecule Array (Simoa) is an ultrasensitive detection technique. The average sensitivity of the Simoa detection method is more than 1,200 times that of the conventional enzyme-linked immunosorbent assay (ELISA), and the coefficient of variation is less than 10% (Kuhle et al., 2016; Wilson et al., 2016), and thus can be used as a quantitative detection method for plasma samples.

In this study, to elucidate the changes in plasma ATN in clinically diagnosed AD patients, the ultrasensitive Simoa detection method was used to measure the expression levels of plasma Aβ42, Aβ40, t-tau, p-tau181, and NfL in patients with mild-to-moderate AD. Concurrently, the expression levels of plasma inflammatory factors (e.g., IL-1β, IL-6, IL-8, and TNF-α.) were examined to evaluate whether there is systemic inflammation in AD patients. Furthermore, we investigated the relationship between plasma inflammatory factors and AD plasma biomarkers to explore whether their combination increases the diagnostic accuracy, and thus provide some basis for the direction of AD treatment.

## Materials and methods

### Study design and participants

All participants aged 50–90 years were recruited through posters at Dongzhimen Hospital, Beijing University of Chinese Medicine from August 2020 to August 2021. All subjects underwent medical history assessment, physical examination, and cognitive function assessment. Participants who met the inclusion criteria were divided into two groups, which included 30 cases in the AD group and 44 cases in the normal control (NC) group. The individuals in the NC group had concerns about memory decline, but were ultimately ruled out by neuropsychological screening. This study was approved by the Ethics Committee of Dongzhimen Hospital and the written consent of the participants was obtained.

### Inclusion and exclusion criteria

The diagnostic criteria for AD dementia are based on the “probable AD dementia” core clinical criteria established by the National Institute on Aging-Alzheimer’s Association (NIA-AA) (McKhann et al., 2011). The evaluation of all subjects was reviewed by a consensus panel consisting of neurologists and geriatricians. The inclusion and exclusion of AD dementia were as follows: (1) history of progressive cognitive decline over 6 months; (2) impairment in more than two cognitive domains including episodic memory impairment; (3) Mini-Mental State Examination (MMSE) scores ≤26 points; (4) impaired activities of daily living (ADL) ≥16 points (Ni et al., 2015); (5) clinical dementia rating global score (CDR-GS) ≥1 point; (6) hippocampal atrophy as described previously (Wei et al., 2019); and (7) cases with vascular disease or other possible causes that may affect cognitive function were excluded.

The NC subjects were identified in accordance with criteria used in the Mayo research study (Ivnik et al., 1992): (1) no active neurological or psychiatric disease; (2) no psychotropic medication; and (3) no medical disorder for which the disorder or its treatment could compromise cognitive function, an MMSE score >26 points (for people attaining higher education), MMSE score >23 points (middle school), MMSE score >22 points (primary school), MMSE score >19 points (Illiteracy), scores based on previous studies of the Chinese population (Shi et al., 2014; Tian et al., 2016); the delayed story recall (DSR) scale was within the normal range (50–64 years old >15.5 points, 65–74 years old >12.5 points, and over 75 years old >10.0 points) (Shi et al., 2014); the ADL scale <16 points (Lawton and Brody, 1969); CDR-GS = 0 (Hughes et al., 1982). Conditions such as anxiety and depression that may have been related to memory complaints were excluded.

### Neuropsychological tests

Comprehensive neuropsychological tests were performed by certified psychometrists. The psychological tests used have been tested for validity in the Chinese population. Each participant received an approximately 60 min cognitive test protocol, including (1) MMSE; (2) ADL; (3) immediate story recall (ISR) and DSR scale; (4) clock drawing test (CDT); (5) trail making test (TMT-A and TMT-B); (6) CDR scale; (7) Hamilton depression scale (HAMD); and (8) Hamilton anxiety scale (HAMA).

### Plasma β-amyloid, tau, and neurodegeneration biomarkers and inflammatory factors measurement

The time interval between blood sample collection and neuropsychological assessment should not exceed 1 week. 5 ml of peripheral venous blood was collected in EDTA anticoagulant tubes when subjects were fasting. Centrifugation at 2,000× *g* for 15 min was completed within 1 h after blood collection. After centrifugation, the plasma samples were stored at −80°C without repeated freeze-thaw cycles. The shortest time interval from sampling to testing was 16 days and the longest was 379 days, the median and the quartiles (25th and 75th) of time interval were 306 (277, 326). Plasma sample concentrations were measured by H-Wayen Biotechnologies (Shanghai, China) using an ultrasensitive Simoa on an HD-X Analyzer (Quanterix, Lexington, MA, United States). The Neuro 3-Plex A Kit (Cat. No. 101995), p-tau181 Advantage V2 Kit (Cat. No. 103714), NF-light Kit (Cat. No. 103186), IL-1β 2.0 Kit (Cat. No. 101605), IL-6 2.0 Kit (Cat. No. 101622), IL-8 Kit (Cat. No. 100198), and TNF-α 2.0 Kit (Cat. No. 101580) were used to measure Amyloid biomarkers (Aβ40, Aβ42), tau biomarkers (tau, p-tau181), neuro-axonal injury biomarker (NfL), and inflammatory factors (IL-1β, IL-6, IL-8, TNF-α). All assays were performed by personnel who were blind to the participants’ information.

### Statistical analyses

For normally distributed continuous variables were described by mean and standard deviation and Student’s *t*-tests were performed. The median and quartile 1 (Q1) to quartile 3 (Q3) were used to describe the skewed distributed continuous variables with Mann–Whitney *U*-test. For categorical variable, number (n), and frequencies (%), Chi-square test was performed. Multiple linear regression was used for association analysis controlling for covariates. Sensitivity and specificity analysis of discrimination was expressed by receiver operating characteristic (ROC) curve and area under the curve (AUC), and the optimal cut-off value was determined by the Youden index. *p* < 0.05 was considered statistically significant. Boxplots and points were used to present the distributions of original values of plasma biomarkers. Scatter plots were used to illustrate the correlations between MMSE, memory, visual space, executive ability and plasma biomarkers. Data were analyzed using IBM SPSS Statistics for Windows, version 25.0 (IBM Corp., Armonk, NY, United States). Box plots were visualized by GraphPad Prism (version 8.0.0^1^). Scatter plots and ROC were produced using the ggplot2 package in R (version 4.0.2).

## Results

### Participants’ characteristics

Seventy-four subjects were included in this study, including 30 in the AD group and 44 in the NC group. There was no difference in age and gender ratio between the two groups, but the AD group had fewer years of education, compared with the NC group. There were significant differences in the MMSE, ISR, DSR, ADL, CDR, TMT-A, TMT-B, and CDT scores between the two groups (*p* < 0.01), but there was no significant difference in the HAMA and HAMD scores between the two groups (*p* > 0.05). The results of cognitive function of the two groups are shown in Table 1.

**TABLE 1:** The characteristics of study participants.

Characteristic	Group		*F*/*Z*/Chi-Square	*p*-Value
	AD	NC		
	(*n* = 30)	(*n* = 44)		
Sex				0.397
Female, *n* (%)	12 (40%)	22 (50%)		
Male, *n* (%)	18 (60%)	22 (50%)		
Age, years	75.27 ± 7.67	71.86 ± 7.76	*F* = 0.001	0.067
Education, years	9(6, 12)	12(9, 15)	*Z* = −2.858	0.004*
MMSE/30	17.5(11.5, 22)	29(28, 30)	*Z* = −7.312	0.00*
DSR/56	0.00(0.00, 3.50)	26.5(20.25, 33.0)	*Z* = −7.260	0.00*
ADL/56	21.5(17.75, 29.50)	14(14, 14)	*Z* = −7.608	0.00*
CDR/3	1.0(1.0, 2.0)	0.0(0.0, 0.0)	*Z* = −8.126	0.00*
TMT-A/150 s	150(99.5, 150)	50(37.25, 70)	*Z* = −5.202	0.00*
TMT-B/300 s	300(176, 300)	114.5(75, 300)	*Z* = −3.592	0.00*
CDT/4	2.0(1.0, 3.25)	4.0(4.0, 4.0)	*Z* = −6.633	0.00*
ISR/56	5.5(0.0, 11.0)	30.0(24.25, 33.75)	*Z* = −6.653	0.00*
HAMD/52	4.0(2.0, 7.0)	2.5(1.0, 6.0)	*Z* = −1.359	0.174
HAMA/56	3.0(1.75, 6.25)	3.0(1.0, 5.0)	*Z* = −1.131	0.258

For normally distributed continuous variables were described by mean and standard deviation and Student’s t-tests were done. The median and quartile 1 (Q1) to quartile 3 (Q3) were used to describe the skewed distributed continuous variables with Mann–Whitney U-test. For categorical variable, number (n) and frequencies (%) was employed, Chi-square test was done. *p < 0.05 compared with normal control group. AD, Alzheimer’s diseases; NC, normal control; MMSE, Mini-Mental Status Examination; ADL, Activity of Daily Living; ISR, Immediate story recall; DSR, delayed Story Recall Scale; CDT, clock drawing test; TMT-A, trail making test A; TMT-B, trail making test B; CDR, Clinical Dementia Rating Scale; HAMD, Hamilton Depression Scale; HAMA, Hamilton Anxiety Scale.

### Plasma β-amyloid, tau, and neurodegeneration biomarkers and inflammatory factors

**FIGURE 1:**
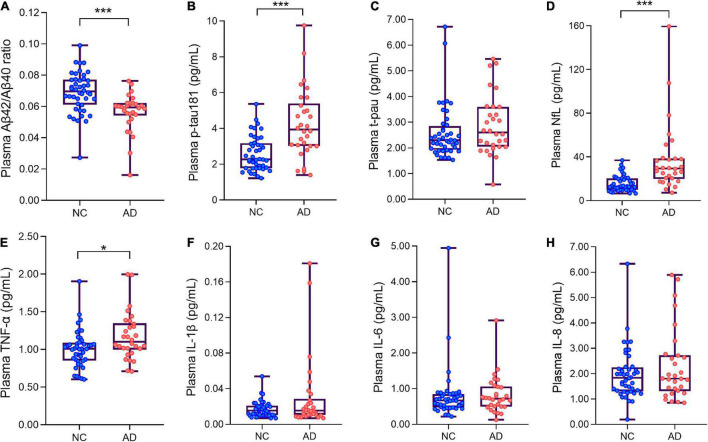
Box plots showing the levels of plasma ANT biomarkers and inflammatory factors of AD patients and normal controls. **(A)** The levels of plasma Aβ42/Aβ40 ratio of AD patients and normal controls. **(B)** The levels of plasma p-tau181 of AD patients and normal controls. **(C)** The levels of plasma t-pau of AD patients and normal controls. **(D)** The levels of plasma neurofilament protein light chain (NfL) of AD patients and normal controls. **(E–H)** Four plasma inflammatory factors (TNF-α, IL-1β, IL-6, IL-8) levels in AD and NC groups. *p < 0.05,***p < 0.001 compared with the normal group. Boxes show interquartile range, the horizontal lines are medians. AD, Alzheimer’s diseases; NC, normal control; pg/ml, picogram/milliliter.

The Aβ42/Aβ40 ratio in the AD group was significantly lower than that in the NC group (*p* < 0.01) (Figure 1A). The plasma level of p-tau181 was significantly higher in the AD group than in the NC group (*p* < 0.01) (Figure 1B), but there was no significant difference in the expression level of t-tau between the two groups (*p* > 0.05) (Figure 1C). The plasma level of NfL was significantly higher in the AD group than in the NC group (*p* < 0.01) (Figure 1D). Among the four inflammatory factors tested (IL-1β, IL-6, IL-8, and TNF-α), only TNF-α showed differences between the groups (*p* < 0.05); the TNF-α concentration in the AD group was higher than that in the NC group (Figures 1E–H and Table 2).

**TABLE 2:** Expression levels of plasma ANT biomarkers and inflammatory factors.

Biomarkers (pg/mL)	Group		F/Z	*p*-Value

	AD	NC		
	(*n* = 30)	(*n* = 44)		
IL-1β	0.016(0.010, 0.029)	0.016(0.01, 0.021)	Z = −0.419	0.675
IL-6	0.72(0.50, 1.06)	0.67(0.48, 0.85)	Z = −0.72	0.471
IL-8	1.81(1.31, 2.73)	1.84(1.31, 2.25)	Z = −0.385	0.700
TNF-α	1.10(1.00,1.35)	1.01(0.85, 1.09)	Z = −2.554	0.011*
NfL	29.46(19.76, 38.62)	12.99(9.90, 20.31)	Z = −4.844	0.000*
t-tau	2.60(2.04, 3.60)	2.30(1.93, 2.85)	Z = −1.343	0.179
p-tau181	3.94(3.02, 5.39)	2.27(1.78, 3.18)	Z = −3.969	0.000*
Aβ40	191.90 ± 36.19	172.08 ± 41.63	F = 0.228	0.038*
Aβ42	10.73 ± 2.67	11.64 ± 2.59	F = 0.166	0.148
Aβ42/Aβ40 ratio	0.06(0.05, 0.06)	0.70(0.06, 0.77)	Z = −3.996	0.000*

For normally distributed continuous variables were described by mean and standard deviation and Student’s t-tests were done. The median and quartile 1 (Q1) to quartile 3 (Q3) were used to describe the skewed distributed continuous variables with Mann–Whitney U-test. For categorical variable, number (n) and frequencies (%) was employed, Chi-square test was done. *p < 0.05 compared with normal control group. AD, Alzheimer’s diseases; NC, normal control; Aβ, amyloid-beta protein; t-tau, total tau; NfL, neurofilament protein light chain; p-tau181, tau phosphorylated at threonine 181; pg/ml, picogram/milliliter.

### Plasma β-amyloid, tau, and neurodegeneration biomarkers and cognitive functions

**FIGURE 2:**
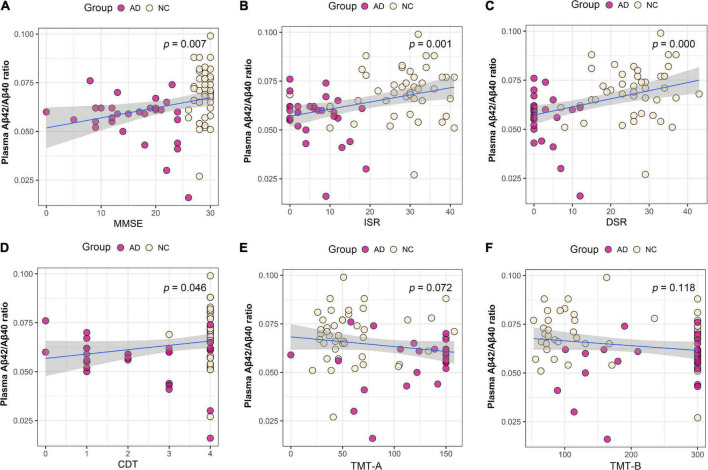
Association between plasma Aβ42/Aβ40 ratio and cognitive domains. **(A)** Association between plasma Aβ42/Aβ40 ratio and MMSE. **(B)** Association between plasma Aβ42/Aβ40 ratio and ISR. **(C)** Association between plasma Aβ42/Aβ40 ratio and DSR. **(D)** Association between plasma Aβ42/Aβ40 ratio and CDT. **(E)** Association between plasma Aβ42/Aβ40 ratio and TMT-A. **(F)** Association between plasma p-tau181 and TMT-B. Multiple regression analysis was used and adjusted for years of education. AD, Alzheimer’s diseases; NC, normal control; MMSE, Mini-Mental Status Examination; ADL, Activity of Daily Living; ISR, Immediate story recall; DSR, delayed Story Recall Scale; CDT, clock drawing test; TMT-A, trail making test A; TMT-B, trail making test B.

**FIGURE 3:**
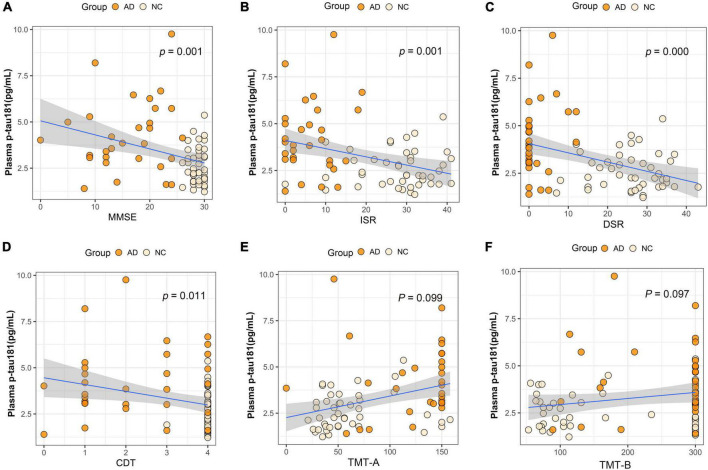
Association between plasma p-tau181 and cognitive domains. **(A)** Association between plasma p-tau181 and MMSE. **(B)** Association between plasma p-tau181 and ISR. **(C)** Association between plasma p-tau181 and DSR. **(D)** Association between plasma p-tau181 and CDT. **(E)** Association between plasma p-tau181 and TMT-A. **(F)** Association between plasma p-tau181 and TMT-B. Multiple regression analysis was used and adjusted for years of education. AD, Alzheimer’s diseases; NC, normal control; MMSE, Mini-Mental Status Examination; ADL, Activity of Daily Living; ISR, Immediate story recall; DSR, delayed Story Recall Scale; CDT, clock drawing test; TMT-A, trail making test A; TMT-B, trail making test B; pg/ml, picogram/milliliter.

**FIGURE 4:**
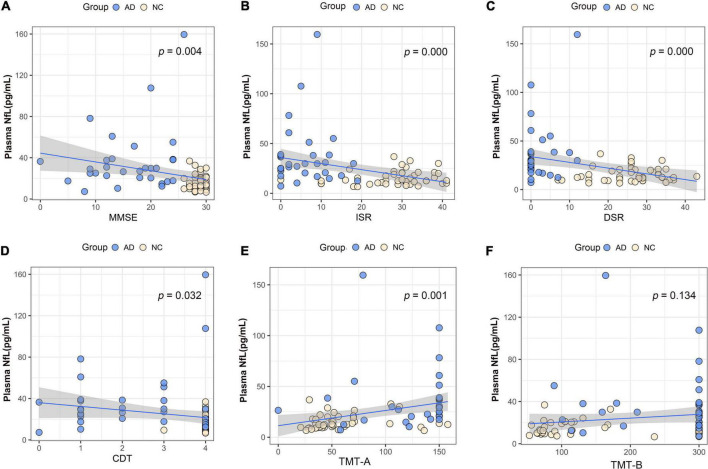
Association between plasma neurofilament protein light chain (NfL) and cognitive domains. **(A)** Association between plasma NfL and MMSE. **(B)** Association between plasma NfL and ISR. **(C)** Association between plasma NfL and DSR. **(D)** Association between plasma NfL and CDT. **(E)** Association between plasma NfL and TMT-A. **(F)** Association between plasma NfL and TMT-B. Multiple regression analysis was used and adjusted for years of education. AD, Alzheimer’s diseases; NC, normal control; MMSE, Mini-Mental Status Examination; ADL, Activity of Daily Living; ISR, Immediate story recall; DSR, delayed Story Recall Scale; CDT, clock drawing test; TMT-A, trail making test A; TMT-B, trail making test B; pg/ml, picogram/milliliter.

**FIGURE 5:**
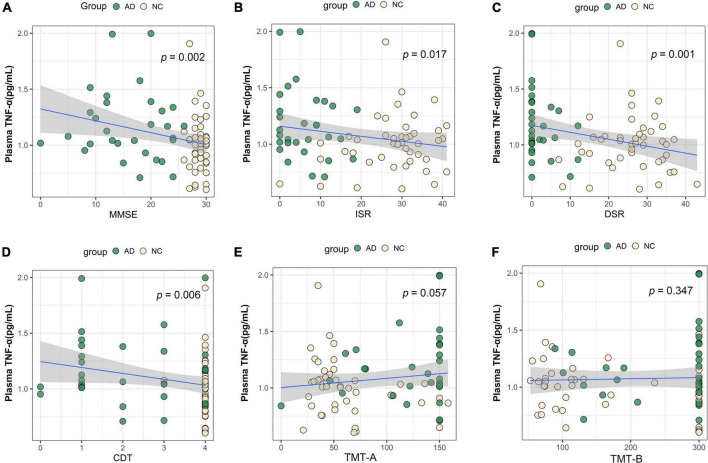
Association between plasma TNF-α and cognitive domains. **(A)** Association between plasma TNF-α and MMSE. **(B)** Association between plasma TNF-α and ISR. **(C)** Association between plasma TNF-α and DSR. **(D)** Association between plasma TNF-α and CDT. **(E)** Association between plasma TNF-α and TMT-A. **(F)** Association between plasma TNF-α and TMT-B. Multiple regression analysis was used and adjusted for years of education. AD, Alzheimer’s diseases; NC, normal control; MMSE, Mini-Mental Status Examination; ADL, Activity of Daily Living; ISR, Immediate story recall; DSR, delayed Story Recall Scale; CDT, clock drawing test; TMT-A, trail making test A; TMT-B, trail making test B; pg/ml, picogram/milliliter.

We performed association analysis of cognitive function with plasma biomarker levels that differed between the groups, using multiple regression analysis adjusted for years of education. The results showed that the plasma Aβ42/Aβ40 ratio and the MMSE (β = 0.293, *p* = 0.007), ISR (β = 0.362, *p* = 0.001), DSR (β = 0.415, *p* = 0.000), and the CDT (β = 0.213, *p* = 0.046) had a positive correlation (Figures 2A–F). Plasma p-tau181 negatively correlated with MMSE (β = −0.342, *p* = 0.001), ISR (β = −0.35, *p* = 0.001), DSR (β = −0.392, *p* = 0.000), and CDT (β = −0.268, *p* = 0.011) (Figures 3A–F). Plasma NfL negatively correlated with MMSE (β = −0.313, *p* = 0.004), ISR (β = −0.384, *p* = 0.000), DSR (β = −0.379, *p* = 0.000), and CDT (β = −0.230, *p* = 0.032) positively correlated with TMT-A (β = 0.352, *p* = 0.001) (Figures 4A–F). Plasma TNF-αnegatively correlated with MMSE (β = −0.339, *p* = 0.002), ISR (β = −0.264, *p* = 0.017), DSR (β = −0.353, *p* = 0.001), and CDT (β = −0.296, *p* = 0.006) (Figures 5A–F).

### Diagnostic performance of plasma β-amyloid, tau, and neurodegeneration biomarkers

**FIGURE 6:**
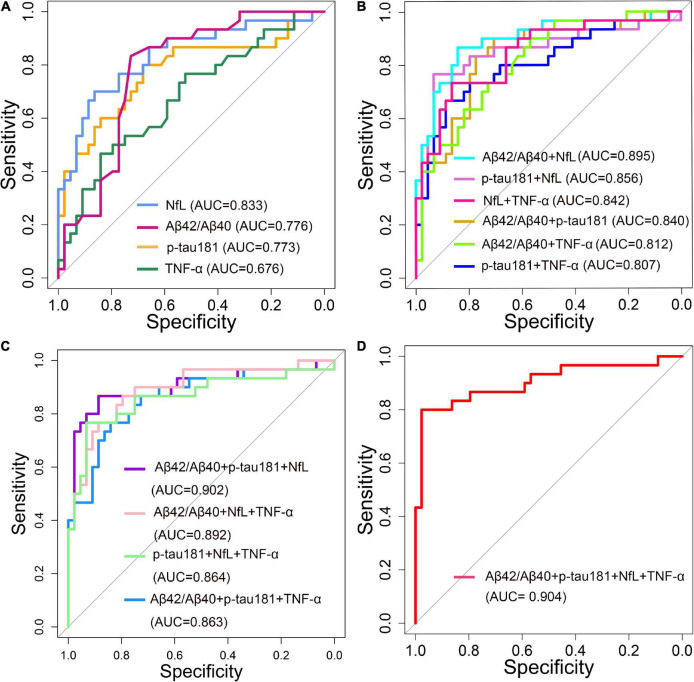
Receiver operating characteristic (ROC) curve analyses of plasma ANT biomarkers and inflammatory factors for distinguishing Alzheimer’s disease (AD) dementia from normal control (NC) of 74 patients (30 AD dementia and 44 NC). **(A)** Single biomarker (Aβ42/Aβ40, p-tau181, NfL and TNF-α) were used as a predictor, respectively. **(B)** Two composite biomarkers (Aβ42/Aβ40 ratio + p-tau181, Aβ42/Aβ40 ratio + NfL, Aβ42/Aβ40 ratio + TNF-α, p-tau181 + NfL, p-tau181 + TNF-α, NfL + TNF-α) were used as a predictor, respectively. **(C)** Three composite biomarkers (Aβ42/Aβ40 ratio + p-tau181 + NfL, Aβ42/Aβ40 ratio + p-tau181 + TNF-α, Aβ42/Aβ40 ratio + NfL + TNF-α, p-tau181 + NfL + TNF-α) were used as a predictor, respectively. **(D)** Four composite biomarkers (Aβ42/Aβ40 ratio + p-tau181 + NfL + TNF-α) were used as a predictor.

The diagnostic performance of plasma ATN biomarkers alone to differentiate AD from NC was acceptable, the discriminant validity was NfL, Aβ42/Aβ40 ratio, and p-tau181 in descending order, and their AUC values were 0.833, 0.776, and 0.773, respectively (Figure 6A). Plasma TNF-α used alone had a low validity of discriminating AD from NC (AUC = 0.676). A combined diagnostic model was used to assess the discriminative validity of the combination of two plasma ATN biomarkers. The diagnostic performance from high to low was Aβ42/Aβ40 ratio + NfL (AUC = 0.895), p-tau181 + NfL (AUC = 0.856), Aβ42/Aβ40 ratio + p-tau181 (AUC = 0.840) (Figure 6B). The combination of three ATN plasma markers (Aβ42/Aβ40 ratio + p-tau181 + NfL) had the best discriminative performance (AUC = 0.902) (Figure 6C), with a sensitivity and specificity of 0.867 and 0.886, respectively. Combining ATN markers with TNF-α did not appear to improve the diagnostics performance (AUC = 0.904) (Figure 6D).

## Discussion

In this study, we compared the plasma levels of Aβ42, Aβ40, p-tau181, t-tau, and NfL in patients with probable AD dementia in mild-to-moderate stages with those in the NC group. The plasma ratio of Aβ42/Aβ40 was significantly decreased in the clinically diagnosed AD group, and the plasma levels of p-tau181 and NfL were significantly increased. T-tau, a biomarker of neurodegeneration, is usually elevated in the CSF, but it not significantly altered in the plasma samples in our study. Regardless of plasma Aβ42/Aβ40 ratio, p-tau181, and NfL levels, when applied alone the ability of these factors to distinguish AD from NC was only moderate (AUC: 0.773–0.833). The results of the diagnostic model showed that the combination of two ATN biomarkers significantly increased the accuracy of distinguishing AD from NC (AUC: 0.807–0.895). Simultaneously incorporating Aβ42/Aβ40 ratio, p-tau, and NfL into the analysis model resulted, in AUC = 0.902. Furthermore, Aβ42/Aβ40 ratio, p-tau181, and NfL were all associated with global cognition function (MMSE) and memory abilities (DSR and ISR). Except for TNF-α, there were no between-group differences in other inflammatory factors (IL-1β, IL-6, and IL-8).

Since the pathological features of AD (senile plaques and neurofibrillary tangles) were first reported in 1907 (Gurwitz, 1997), definite AD diagnosis has relied on postmortem autopsy. Based on technological advances in the detection of molecules that reflect AD pathology, the NIA-AA research framework proposed a biological definition and classification of AD (Jack et al., 2018). AD pathology can be reliably detected *in vivo* using PET or CSF testing, but the inconsistency rate between PET and CSF results is about 10–20% in the same individuals (de Wilde et al., 2019). However, many patients do not undergo invasive CSF testing and expensive PET scans because of concerns about lack of treatment options, iatrogenic harm, and high costs. The undiagnosed rate of AD in China is higher than that in developed countries, with the pooled rate of undetected dementia being 61.7% (95% CI 55.0–68.0%) (Lang et al., 2017), and most of these cases are AD patients.

Blood samples are relatively easy to collect and can be collected multiple times, hence plasma AD biomarker measurements are more accessible than CSF testing and PET scans. The plasma Aβ level is 0.1–0.2 of the level found in the CSF, Furthermore, the plasma contains high levels of interfering factors, making it difficult to obtain stable and consistent results with commonly used ELISA platforms (Lue et al., 2017). With the application of ultrasensitive immunoassays, the level of AD biomarkers in the plasma can be more reliably determined (Wilson et al., 2016). Simoa is a double-antibody sandwich ELISA. Compared with traditional ELISA, Simoa technology, which can detect single molecules, can determine very low protein levels.

A study by Brickman et al. (2021), which used the Simoa method to measure plasma ATN biomarkers in a population clinically diagnosed with AD, found differences in the level of p-tau but not in t-tau, which is consistent with our findings. In contrast, their study did not find differences in the Aβ42/Aβ40 ratio and NfL concentration between the AD group and the control group. Another study confirmed increased concentrations of plasma NfL in patients with AD compared with non-demented controls, which is consistent with our findings (Lewczuk et al., 2018). A longitudinal cohort study findings suggest that plasma NfL can be used as a non-invasive biomarker to track neurodegeneration in patients with AD (Mattsson et al., 2019). As a marker of axonal damage, NfL has received increasing attention in recent years. Studies have found that the increase in the plasma NfL level is related to the decline in cognitive function (Illán-Gala et al., 2021). Plasma NfL can be used as a non-invasive biomarker of neurodegeneration in AD patients, and the faster the NfL level rises, the faster the deterioration of overall cognitive performance (Brosseron et al., 2014). Some studies suggest that NfL is not only elevated in AD, but also in other types of dementia. Although not specific, plasma NfL levels are helpful in detecting AD progression (Rauchmann et al., 2021). Unlike other biomarkers, plasma p-tau181 showed good agreement among the results of different studies. Plasma p-tau181 has been proposed to be a specific marker for tau pathology in AD. Recent studies supported its use as a screening tool by showing increased levels in patients with AD compared with controls or patients with other tauopathies (Tijms and Teunissen, 2021). Elevated levels of tau reflect neuronal damage, and studies have shown that elevated levels of t-tau and p-tau181 in the CSF reflect degenerative processes in cortical regions typically involved in AD, however, p-tau181 associated with neurodegenerative changes in early AD may be more specific (Thomann et al., 2009).

Our results showed an association of plasma markers with cognitive function. When the entire data were analyzed, our results showed that the plasma Aβ42/Aβ40 ratio p-tau181, NfL, and TNF-α levels were all associated with impairments in global cognition (MMSE), memory (ISR and DSR), and visuospatial abilities (CDT) but not withe executive function (TMT-A and TMT-B), only plasma NfL was associated with TMT-A. However, data from the AD group did not show an association of plasma markers with cognitive function (Supplementary Table 1). Previous cross-sectional data have not reached consistent conclusions about the association of plasma biomarkers with different domains of cognitive impairment. Chatterjee et al.’s (2018) research found that plasma NfL concentrations inversely correlated with verbal and visual episodic memory, working memory and executive function and global cognition. Rauchmann et al. (2021) concluded that cognitive performance was associated cross-sectionally with NfL in all ATN subgroups, and with p-tau181 only in AD spectrum individuals. A study conducted in a Chinese population also showed that plasma Aβ42/Aβ40 ratio, p-tau181, and NfL were all associated with different cognitive domains, but plasma p-tau181 was associated with a wider range of cognitive domains, followed by NfL (Xiao et al., 2021). Whether in demented or non-demented elderly individuals, NfL as a downstream factor seems to be a relatively stable plasma marker that can reflect the cognitive function of the subjects (He et al., 2021). Although Aβ42 as an upstream factor is commonly used to distinguish AD from non-AD, longitudinal studies have only shown a weak association between cognitive decline and amyloid burden (Villemagne et al., 2011). Differences in the results of the overall data and the AD subgroup data suggest that cognitive function and AD pathological progression may have different time-course profiles.

The results of plasma ATN biomarkers and CSF markers are not always consistent, which may reflect different metabolic pathways. In this study, we did not collect CSF markers for comparison, nor did we conduct subgroup analysis of the most common genetic factors, such as APOE ε4 status. These more in-depth discussions may provide a better basis for clinical application of plasma ATN biomarkers. In addition, limited by the current sample size, we did not perform subgroup analysis according to the severity of AD dementia.

Systemic inflammation may be associated with AD pathogenesis, but this idea remains controversial. Based on a literature review investigating the relationship between inflammation and AD, we measured the plasma levels of four inflammatory factors (IL-1β, IL-6, IL-8, and TNF-α). Except that the plasma concentration of TNF-α that was elevated in the AD group, no group differences were found for the other three inflammatory factors (IL-1β, IL-6, and IL-8). In this study, TNT-α did not significantly improve the performance of the diagnostic model. Holmes et al. (2011) found an increase in the serum levels of proinflammatory cytokine TNF-α in mild and moderate AD patients, which was associated with a two-fold increase in the rate of cognitive decline over a 6-month period. However, in a study by Julian et al. (2018), no correlation between the plasma value of IL-1β, IL-6, TNF-α at diagnosis and cognitive decline during the 2 years of follow-up was found. Chronic systemic inflammation has broad and non- specific effects, and the levels of inflammatory cytokines can be influenced by many factors, which may explain the differences in results across study populations, hence further research is needed.

## Conclusion

The level of plasma ATN biomarkers (Aβ42/40 ratio, p-tua181, and NfL) were significantly changed in clinically diagnosed AD patients and they all correlated with different domains of cognitive impairment. Plasma ATN biomarkers better differentiate mild-to-moderate AD dementia from NC when they are incorporated into diagnostic models together rather than applied individually. However, the expression of plasma inflammatory factors in AD patients requires further research.

## Data availability statement

The data supporting the conclusions of this article will be made available by the authors upon formal and reasonable request.

## Ethics statement

The studies involving human participants were reviewed and approved by the Ethics Committee of Dongzhimen Hospital. The patients/participants provided their written informed consent to participate in this study.

## Author contributions

Q-LS wrote the original draft and curated data. J-NN wrote and edited the manuscript. M-QW, S-WL, TL, D-SF, and TL conducted formal analyses and reviewed the manuscript. JS and J-ZT designed and directed this study. All authors contributed to the article and approved the submitted version.

## Footnotes

1
www.graphpad
