# Supplementary material for: Plasma β-amyloid, tau, neurodegeneration biomarkers and inflammatory factors of probable Alzheimer’s disease dementia in Chinese individuals
Source: Front Aging Neurosci. 2022 Aug 18;14:963845. doi: 10.3389/fnagi.2022.963845 (PMC9433929; doi:10.3389/fnagi.2022.963845)
Supplement: Supplementary file 1 [file Table_1.docx]

**Supplementary table 1.** Association between plasma ATN biomarkers and cognitive domains in AD group.

| **Plasma ATN biomarkers** | **ISR** | | **DSR** | |
| --- | --- | --- | --- | --- |
|  | *r* | *p* | *r* | *p* |
| Aβ42/Aβ40 ratio | -0.24 | 0.201 | -0.236 | 0.209 |
| p-tau181 | 0.122 | 0.522 | 0.266 | 0.155 |
| NfL | -0.002 | 0.992 | 0.123 | 0.518 |
| TNF-α | -0.151 | 0.425 | -0.221 | 0.241 |

Pearson and Spearman analysis were used for correlation analysis, *p* < 0.05 was considered statistically significant. Aβ, amyloid-beta protein; t-tau, total tau; NfL, neurofilament protein light chain; p-tau181, tau phosphorylated at threonine 181; ISR, Immediate story recall; DSR, delayed Story Recall Scale.
